# Light-Triggered Adhesive Silk-Based Film for Effective Photodynamic Antibacterial Therapy and Rapid Hemostasis

**DOI:** 10.3389/fbioe.2021.820434

**Published:** 2022-01-11

**Authors:** Tingting Huang, Zhihao Zhou, Qiaoyuan Li, Xiaoxuan Tang, Xiaoli Chen, Yifan Ge, Jue Ling

**Affiliations:** ^1^ Key Laboratory of Neuroregeneration, Ministry of Education and Jiangsu Province, Co-innovation Center of Neuroregeneration, Jiangsu Clinical Medicine Center of Tissue Engineering and Nerve Injury Repair, Nantong University, Nantong, China; ^2^ Medical School of Nantong University, Nantong University, Nantong, China

**Keywords:** hemostasis, antibacterial activity, reactive oxygen species, silk fibroin, photodynamic therapy

## Abstract

Successful control of massive hemorrhage in deep wounds with irregular shape and low elasticity still remains great challenges in the clinic. As the wound sites are usually at risk of bacterial infection, it is necessary to design an ideal hemostatic agent with rapid hemostasis and excellent antibacterial activity. In this study, we developed a light responsive hemostatic film for effective handling of liver bleeding with promising photodynamic therapy against *S. aureus* onnear infrared (NIR) irradiation. Based on silk fibroin, the film exhibited desirable biocompatibility and mechanical property as a hemostat tape. Significantly, the film tape achieved excellent tissue adhesion and hemostasis *in vivo* within 2 min of UV exposure, which would have a great potential as a multifunctional biomedical material in the field of tissue repair such as wound healing, bone repair, and nerve regeneration.

## Introduction

Massive hemorrhage caused by trauma, traffic accidents, and surgery can lead to excessive blood loss and even death if the effective control of bleeding is not in time (Han et al., 2020; Qiao et al., 2021; Roy et al., 2021). Commercial hemostats, such as gauze, gelatin sponge, and bandages, have been considered to be highly effective in handling bleeding by sealing the wound surface of the bleeding site (Arnaud et al., 2008; Lan et al., 2015; Vilardo et al., 2017). However, they are often invalid for deep wounds with irregular shape and low elasticity, such as the liver, brittle tissue, and abundant capillaries, which are inconvenient to press. Meanwhile, the injury sites are usually at high risk of bacterial infection, which can result in serious symptoms (Deng et al., 2021; Zheng et al., 2021; Zhu et al., 2021). Therefore, it is necessary to develop multifunctional nonpressing hemostatic agents that can perform rapid hemostasis with effective antibacterial activity for minimizing blood loss and improving survival in the clinic.

Silk fibroin (SF) is a natural protein from the silk cocoon with tunable mechanical strength and low immunogenicity (Kim et al., 2021). It is considered as a bioactive and biocompatible protein biomaterial that is suitable for various biomedical applications, such as wound healing, bone repair, and nerve regeneration (Tang et al., 2021a; Bigham et al., 2021; Tang et al., 2021b; Gu et al., 2021; Li et al., 2021). Especially for hemostasis, it has been reported that SF can bind with fibrinogen and blood platelets to facilitate the clotting cascade (Chouhan and Mandal, 2020). Importantly, the hemostatic activity of SF can further act as a cargo to release inflammatory factors to lead hemostasis to the next phase of the healing process (Santin et al., 1999). However, lack of antibacterial activity limits the further application of SF on handling bleeding and dressing the wounds (Xuan et al., 2020).

Recently, as the extensive use of antibiotics causes the emergence of antibiotic-resistant bacteria, photodynamic therapy (PDT) has been regarded as an effective method to kill multidrug-resistant bacteria, such as *Staphylococcus aureus* (*S. aureus*) (Mao et al., 2020). By generating reactive oxygen species (ROS) under light exposure, PDT can kill *S. aureus* in a highly spatiotemporal manner, which prevents abscess recurrence with minimal invasion (Zhou et al., 2020). However, rapid clearance and poor biocompatibility of photodynamic agents limits the efficacy of PDT on treating bacterial infection. Currently, conjugation of photodynamic agents with biocompatible polymers has been considered as a promising strategy to improve efficacy of PDT (Kim et al., 2019; Wang et al., 2020).

**FIGURE 1:**
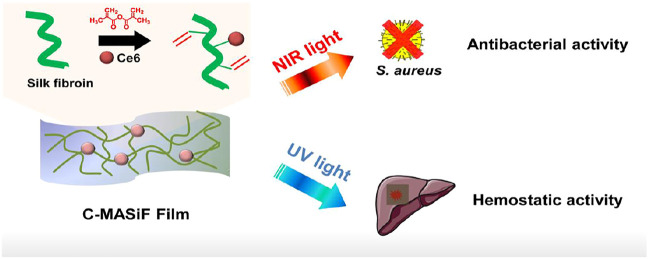
Schematic illustration of silk-based film with effective photodynamic antibacterial therapy and rapid hemostatic activity for handling liver bleeding.

In this study, we developed a silk fibroin based antibacterial film as a rapid hemostatic agent for treating hemorrhage in deep wounds. By conjugating photodynamic agent (Chlorin e6) on methacrylated silk fibroin, C-MASiF film exhibited high toxicity against *S. aureus* under near infrared (NIR) irradiation for treating infection. Importantly, this film achieved excellent *in vivo* hemostasis for handling liver bleeding within 2 min by UV exposure as a desirable hemostatic agent (Figure 1).

## Materials and Methods

### Preparation of MASiF Film

There was 50 g of natural silk (Nantong xianjida cocoon and Silk Fabric Co., Ltd., China) boiled in 2 L of 0.2% w/v sodium carbonate (Xilong Chemical Co., Ltd., China) solution for 30 min at 100°C and dried in the air at room temperature. Then, silk was dissolved in 9.3 M LiBr (Shanghai meiruier Chemical Technology Co., Ltd., China) solution to 20% w/v silk fibroin solution and stirred at 60°C for 3 h and then dialyzed in ultrapure water for 3 days. Then, after concentrated to 10% w/v, 400 µL of silk fibroin solution was added dropwise onto a 2.2 cm × 2.2 cm square glass slide and dried in air to obtain MASiF film. After dried in the air, the film was soaked in absolute ethanol for enhancing β-folding of silk fibroin protein.

### Preparation of C-MASiF Film

Chlorin e6 (Ce6, APExBIO, United States) was activated in a solution containing N-Hydroxysuccinimide (NHS, 98%, Sigma-Aldrich) and 1-(3-Dimethylaminopropyl)-3-ethylcarbodiimde hydrochloride (EDC, Ailan, China) for 2 h. The ratio of Ce6/NHS/EDC was 1:6:6. Then the activated Ce6 solution was slowly added dropwise to the above-mentioned methacrylic acid silk fibroin at a ratio of 1:100 and reacted for 8 h in the dark. The mixture was than dialyzed in the dark for 2 days and concentrated to 15% solution and added onto a 2.2 cm × 2.2 cm square glass slide to obtain MASiF film. After dried in the air, the film was soaked in absolute ethanol for enhancing β-folding of silk fibroin protein.

### Mechanical Performance Test

The stress and strain data were recorded for testing tensile strength of dry films with a constant crosshead separation velocity at 50 mm/min at room temperature.

### Contact Angle

The angle between water droplets and the surface of the film was measured using a contact angle meter (JYPHa, Chengde, China).

### Measurement of Singlet Oxygen Generation

SF film, MASiF film, and C-MASiF film were placed in Singlet Oxygen Sensor Green Reagent (SOSG Meilunbio, Dalian, China) solutions, respectively, and irradiated under NIR laser (20 mW cm^−2^). The fluorescence intensity of the solutions was measured.

### Cytocompatibility

The films were placed in a 24-well plate and then L929 cells were seeded onto the films at a density of 3 × 10^4^ cells/mL in DMEM (Gibco, United States), and incubated at 37°C for 3 days. A live/dead cytotoxicity kit (Molecular Probes, United States) was utilized to visualize the cell behavior. The cytotoxicity of the films was assessed by the cell counting kit-8 (CCK-8) (Beyotime Biotechnology, China).

### *In vitro* Antibacterial Activity

A total of 100 µL of *S. aureus* (10^4^ CFU ml^−1^) were incubated with films at 37°C for 20 min and then irradiated under NIR light (20 mW cm^−2^) for 10 min. *S. aureus* on films was resuspended using 900 µL of phosphate-buffered saline (PBS). Then, 100 µL of the resuspended *S. aureus* solution was spread on solid LB agar plate for 24 h of incubation. Bacterial kill rate: Kill% = C_0_ − C/C_0_ × 100% (where C is the CFU of the experimental group and C_0_ is the CFU of the control group inoculated on a blank plate and without irradiation.)

### Hemolysis Test

A total of 0.005 g/ml of the film’s pieces were dispersed in saline at 37°C for 30 min, respectively. Then fresh anticoagulant rabbit blood was diluted with dispersion liquids at 37°C and the mixture was incubated for 1 h. After centrifugation, the absorbance of the supernatant was measured at 545 nm with a spectrophotometer. Pure saline was used as a negative control, and ultrapure water was used as a positive control. The calculation formula of the hemolysis rate is as follows: HR%=(A-A_negative_)/(A_positive_-A_negative_) × 100%.

### *In vivo* Hemostatic Performance

ICR mice (18–20 g) were first anesthetized and the liver was exposed. Then the liver trauma model was established by puncturing the liver with a syringe. Then, the wound site was covered by MASiF or C-MASiF films with photoinitiator solution (0.5% w/v) and irradiated under UV light for 2 min. The pre-weighted filter paper was placed on the wound for recording the amount of bleeding.

## Results and Discussion

### Fabrication and Characterization of Films

**FIGURE 2:**
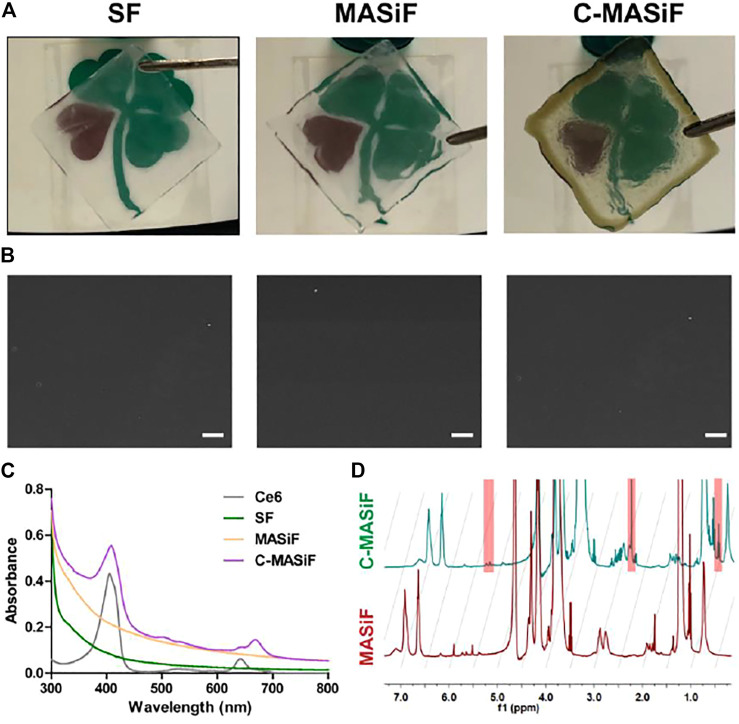
Characterization of MASiF and C-MASiF film. **(A)** Photographs of SF, MASiF, and C-MASiF film. **(B)** SEM images of SF, MASiF, and C-MASiF film. Scale bar: 20 µm. **(C)** UV–vis spectra of Ce6, SF, MASiF, and C-MASiF. **(D)** 1H NMR spectra of MASiF and C-MASiF.

The MASiF and C-MASif films were successfully fabricated and Figure 2A displays that both films were transparent and C-MASif film possessed dark color, due to Ce6 moiety on silk fibroin chains. The images of scanning electron microscopy (SEM) in Figure 2B show that all the films have uniformly dense and smooth surfaces. As shown in Figure 2C, the UV–vis spectrum of C-MASif demonstrated that the characteristic peaks of Ce6 moiety appeared at 400 and at 640 nm. Furthermore, ^1^H NMR spectra of MASiF and C-MASif shows that the characteristic peaks of methacrylate vinyl group were at δ ≈ 6.2 and 5.8, and Ce6 moiety was at δ ≈ 2.8 and 1.1 ppm. These results indicate the successful modification of Ce6 on silk fibroin.

### Hydrophilicity, Mechanical Property, and ROS Production

**FIGURE 3:**
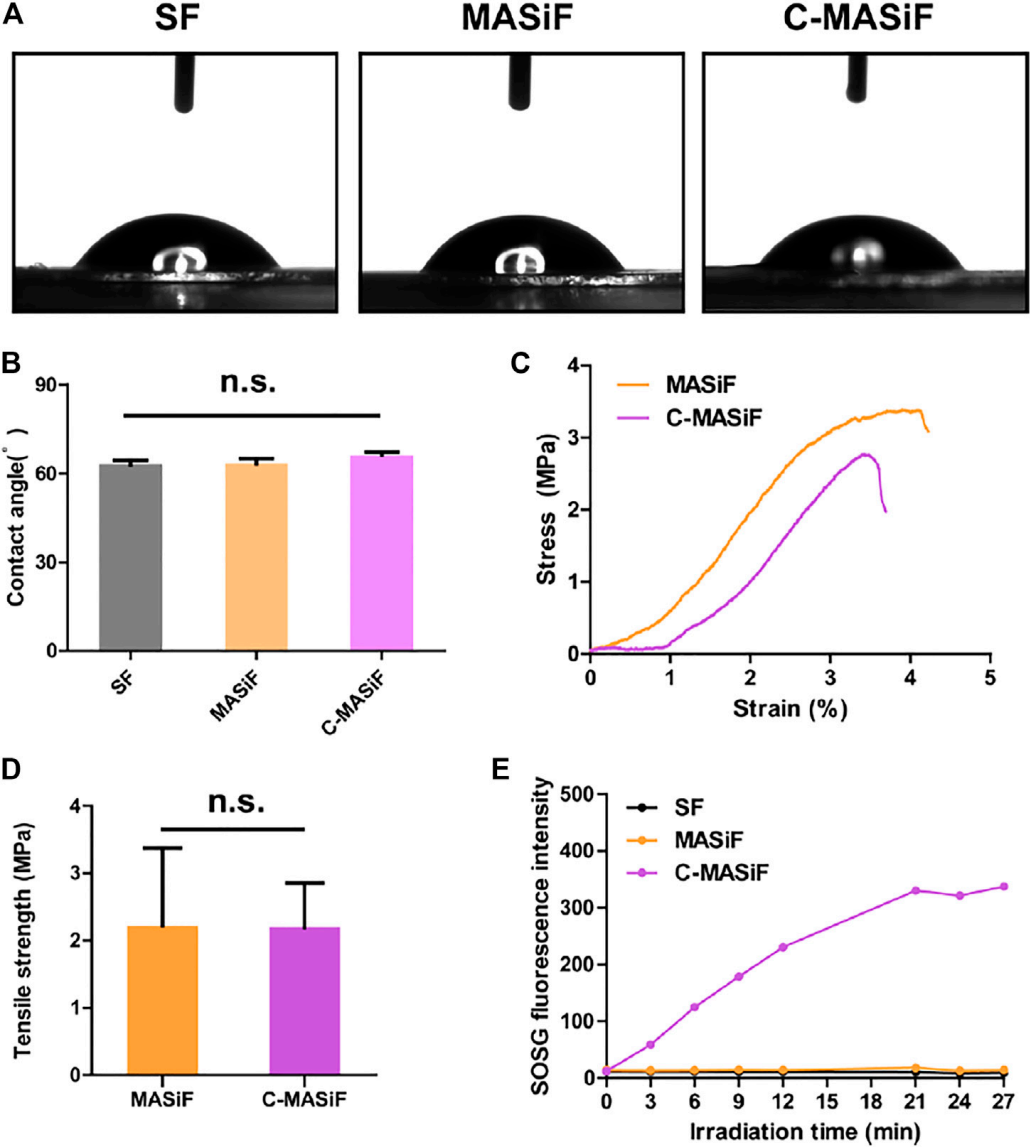
Hydrophilicity and mechanical property of MASiF and C-MASiF film and ROS production by PDT. **(A)** and **(B)** Contact angle of MASiF and C-MASiF film. **(C)** and **(D)** Mechanical property of MASiF and C-MASiF film. **(E)** Fluorescence intensity of SOSG as the functional of irradiation time.

Next, as surface wettability reveals the hydrophilicity and biocompatibility of biomedical materials (Tang et al., 2020), the wettability of the films was evaluated by measuring the contact angle (θ) of water droplets on films. As shown in Figures 3A,B, the contact angles of both MASiF and C-MASif films were less than 90°, indicating the good hydrophilicity of the films. Then, the mechanical property of the films was evaluated using a tensile test. The results show that the average tensile strength of the MASiF and C-MASiF films are more than 2 MPa and there was no statistical difference in the tensile strength of the film (Figures 3C,D), demonstrating that the addition of Ce6 moiety has very limited effect on film tensile strength. In order to exam the efficiency of light-triggered ROS production, the amount of singlet oxygen produced by the films under NIR irradiation (20 mW cm^−2^) was measured by singlet oxygen sensor reagent (SOSG). Upon irradiation of 660 nm light (20 mW cm^−2^), the fluorescence intensity in C-MASiF group enhanced gradually within 20 min, whereas no fluorescence enhancement was obtained for SF or MASiF group, showing that C-MASiF film can effectively produce ROS under NIR light (Figure 3E).

### Cytocompatibility

**FIGURE 4:**
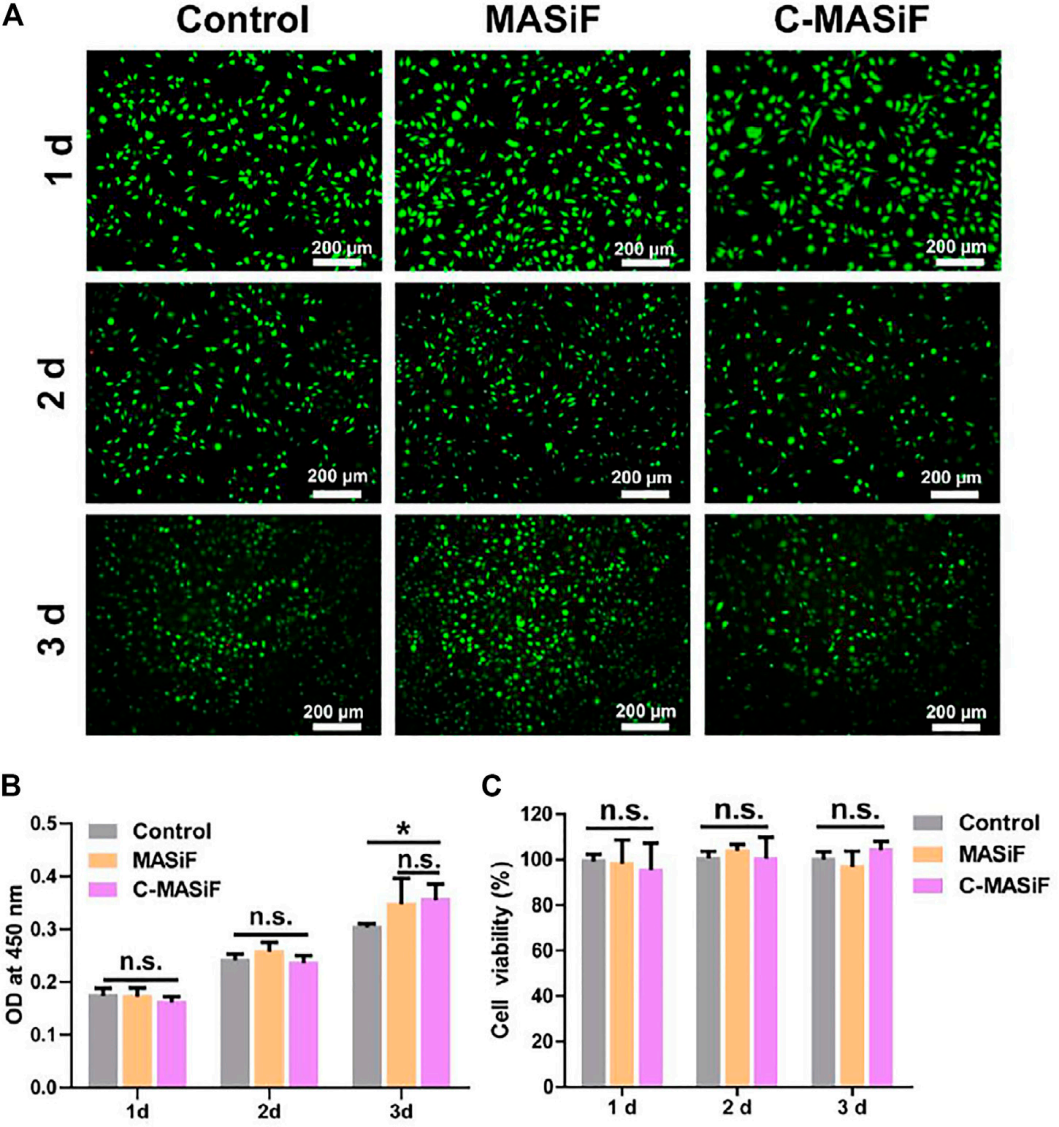
Cytocompatibility of MASiF and C-MASiF film. **(A)** Live/dead assay of L929 cells. **(B)** and **(C)** CCK8 assays of L929 cells. Values represent means ± S.D. (*n* = 3). *p < 0.05.

Fine cytocompatibility is essential for a well-designed biomaterial (Chen et al., 2021; Hao et al., 2021; Ji et al., 2021). Therefore, to evaluate the biocompatibility of films *in vitro*, L929 cells were cultured on the films for 3 days and assessed via live/dead staining and CCK-8 assay. As shown in Figure 4A, by staining with a live/dead cytotoxicity kit, there were very few dead cells found on both MASiF and C-MASif films after 3 days of incubation, indicating the good biocompatibility of these films. Meanwhile, the L929 cells cultured on the films exhibited normal cell proliferation during 3 days of incubation and cell viability were more than 95% on both films (Figures 4B,C).

### Photodynamic Antibacterial Activity of Films

**FIGURE 5:**
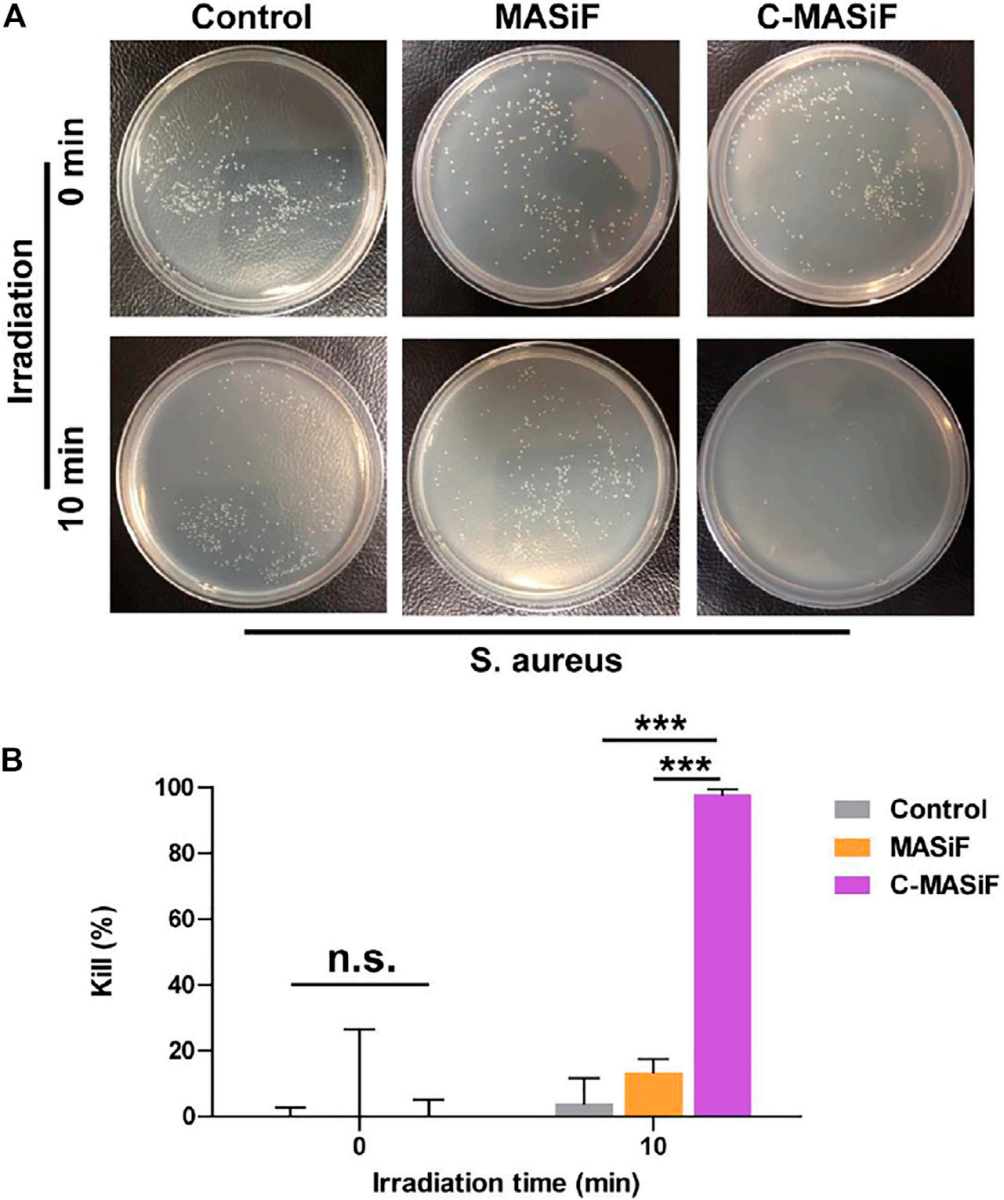
*In vitro* photodynamic antibacterial property of MASiF and C-MASiF films. **(A)** Photographs of *S. aureus* formed on LB-agar plates after PDT. **(B)** Antibacterial efficiency of MASiF and C-MASiF films with NIR irradiation. Values represent means ± S.D. (*n* = 3). ***p < 0.001.

Ideal wound dressings should possess promising antibacterial activity to prevent infection on the wound site (Li et al., 2020; Wang et al., 2021). Therefore, the photodynamic antibacterial activity of the films against *S. aureus* was evaluated. As shown in Figures 5A,B, more than 90% of *S. aureus* was killed on C-MASiF film within 10 min of NIR irradiation (20 mW cm^−2^), due to the effective production of ROS by Ce6 moiety. However, MASiF had poor antibacterial effect on *S. aureus*. Meanwhile, very limited antibacterial effect against *S. aureus* was obtained for the blank plate (the control group) even after 10 min of NIR irradiation (20 mW cm^−2^), illustrating poor antibacterial property of weak NIR laser. These results demonstrated that the C-MASiF film possesses broader potential applications in fields of tissue engineering.

### Hemocompatibility and Tissue Adhesion

**FIGURE 6:**
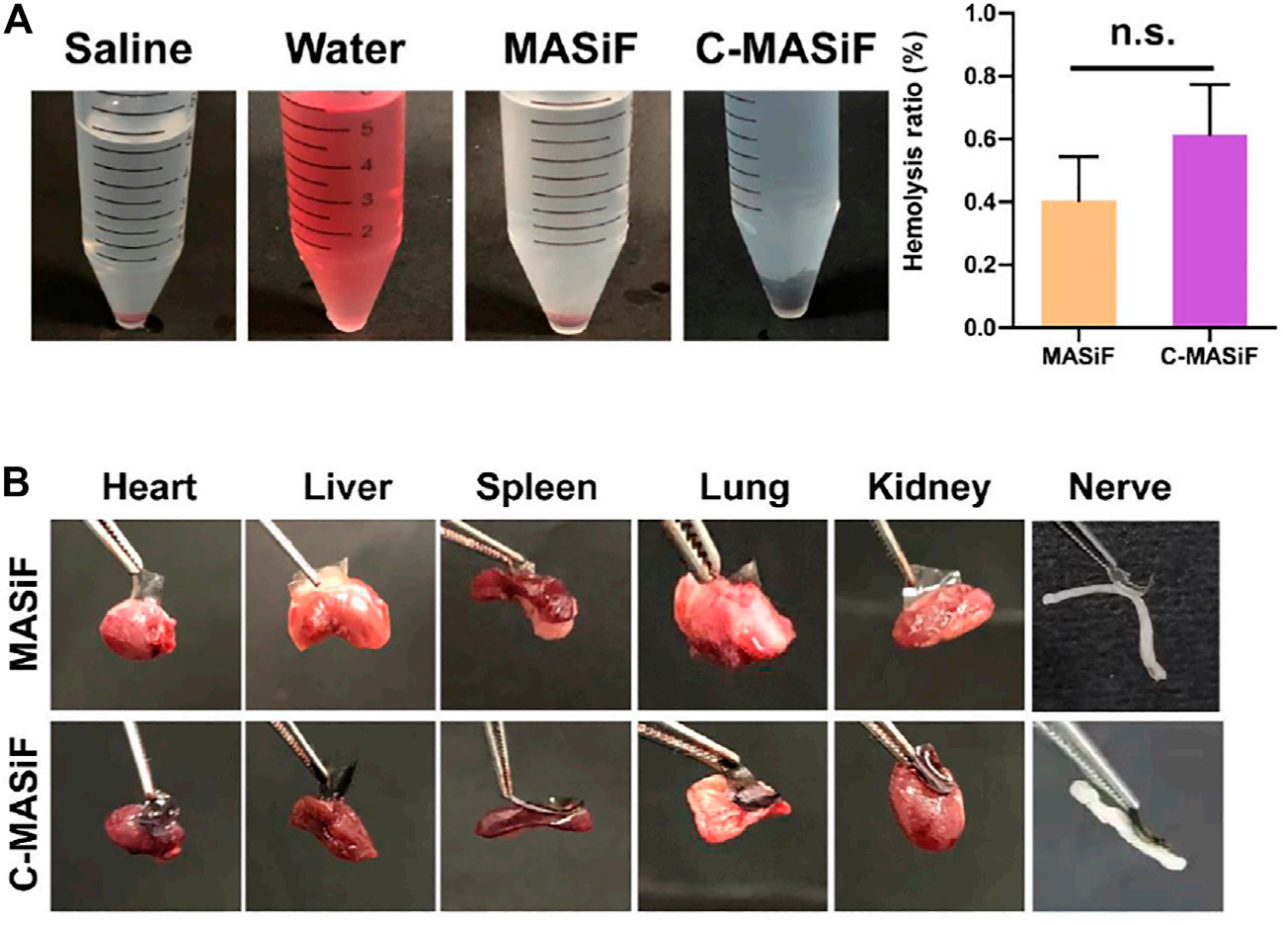
Hemocompatibility and tissue adhesion property of MASiF and C-MASiF films. **(A)** Photographs of MASiF and C-MASiF films incubated with the erythrocytes and hemolysis ratio. Values represent means ± S.D. (*n* = 3). **(B)** Photographs of MASiF and C-MASiF films adhering to various tissues.

Good hemocompatibility is essential for hemostatic agents or wound dressings (He et al., 2020; Yin et al., 2021). The hemocompatibility of the films was evaluated by *in vitro* hemolytic activity assay. The images of solution in Figure 6A showed that liquids in both MASiF and C-MASiF groups had similar color to the saline group. All MASiF and C-MASiF films showed a hemolysis ratio of less than 1%, suggesting that the MASiF and C-MASiF films possess good blood compatibility. Then, the tissue adhesion property of the films to various tissues was evaluated, as it is important for wound dressings and hemostats. As shown in Figure 6B, MASiF and C-MASiF films containing 0.5% of photoinitiator exhibited strong adhesive ability to various tissues, including the heart, liver, spleen, lung, kidney, and nerve tissue, irradiated under UV light (30 mW cm^−2^) for 5 min, due to the photo-chemistry reaction of sulfhydryl groups on tissue with double bonds on films.

### Liver Hemostasis *in vivo*

**FIGURE 7:**
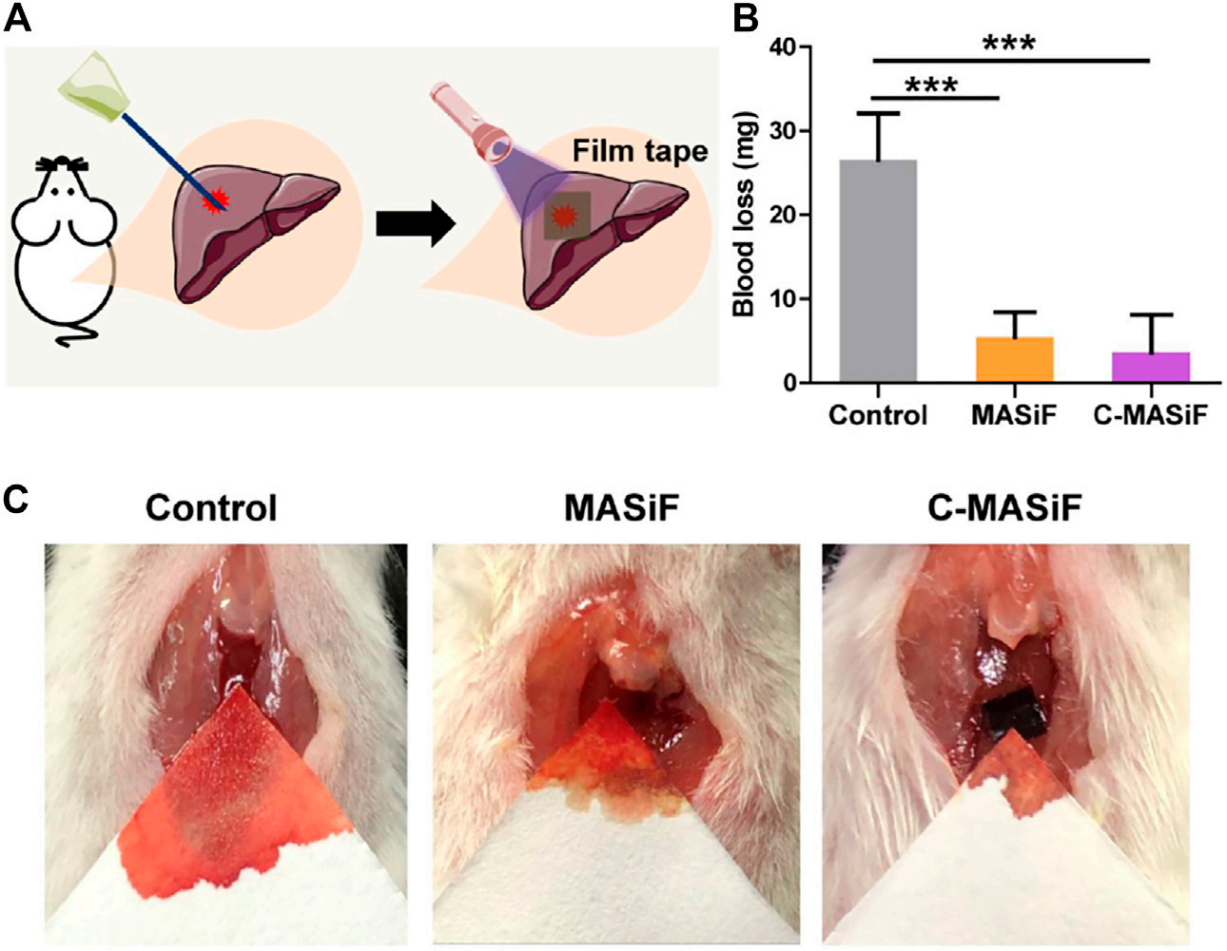
Liver hemostasis *in vivo*. **(A)** Schematic diagram of liver hemostasis process. **(B)** Blood loss of MASiF and C-MASiF films. Values represent means ± S.D. (*n* = 5). ***p < 0.001. **(C)** Photographs of liver hemostasis.

To further evaluate the hemostatic capacity of the films *in vivo*, a mouse liver trauma model was established. ICR mice were anesthetized, and the liver was punctured with a syringe. All the films were soaked in absolute ethanol to obtain β-folding of silk fibroin protein for enhancing their stability for *in vivo* applications in hemostasis. Then MASiF and C-MASiF films containing 0.5% of photoinitiator were applied to the bleeding site with UV irradiation (Figure 7A). The blood loss of the mice in the control group (without any treatment) was 26.5 mg. In contrast, the mice in the MASiF and C-MASiF group showed significantly less blood loss after 2 min of UV irradiation on films, which were 5.5 and 3.7 mg, respectively. The representative photographs in Figure 7C shows the bleeding volume in MASiF and C-MASiF groups was much lower than that in the control group. These results indicate that MASiF and C-MASiF film had a desirable hemostatic effect, due to excellent binding property of SF with fibrinogen and blood platelets to facilitate the clotting and the rapid photo-chemistry reaction of sulfhydryl groups on tissue with double bonds on films.

## Conclusion

In summary, we designed a multifunctional silk fibroin film with rapid light-triggered hemostatic effect and excellent antibacterial performance against *S. aureus* for sealing the wound bleeding and preventing infection. The hemostatic film held good mechanical and biocompatibility. Moreover, the film also exhibited strong tissue adhesion to various biological tissues upon UV light and achieved successful liver hemostasis *in vivo*. Thereby, we believe that this silk-based hemostatic film would have great potential for applications in hemostasis, wound dressings, and tissue repair.

## Data Availability

The raw data supporting the conclusion of this article will be made available by the authors, without undue reservation.
